# The pitfall of pulse pressure variation in the cardiac dysfunction condition

**DOI:** 10.1186/s13054-015-0962-1

**Published:** 2015-06-10

**Authors:** Huai-wu He, Da-wei Liu

**Affiliations:** Department of Critical Care Medicine, Peking Union Medical College Hospital, Chinese Academy of Medical Sciences, 1 Shuaifuyuan, Dongcheng District, Beijing 100730 China

We read with interest the recent articles in *Critical Care* about the limitations of pulse pressure variation (PPV) for predicting fluid responsiveness [1, 2]. We believe that cardiac dysfunction should be included in this list of PPV limitations.

During right ventricular (RV) dysfunction, the inspiration would increase RV afterload and lead to a decrease in RV ejection during mechanical ventilation. Thus, a high PPV is due to afterload variation, and the RV dysfunction would result in a false-positive PPV. Studies had suggested that the evaluation of RV function was important when determining the predictability of PPV [3, 4]. During left ventricular (LV) dysfunction, the increase in pleural pressure that facilitates LV ejection is more pronounced (afterload reduction). Thus, the effect of squeezing the pulmonary blood volume during early inspiration on the LV ejection is amplified and is defined as the dUp. In other words, a high PPV may be due to dUp variation, which would result in a false-positive PPV. Tavernier and colleagues [5] found that the prominence of dUp and absence of dDown might suggest hypervolemia and cardiac contraction dysfunction.

However, Biais and colleagues [2] did not present data for cardiac function in their study. Moreover, cardiac dysfunction is common in critically ill patients. We inferred that this could become a confounding factor for the outcome. Hence, it is worth paying attention to the pitfall of PPV in critically ill patients with cardiac dysfunction.

## Abbreviations

LV: Left ventricular
PPV: Pulse pressure variation
RV: Right ventricular

## References

[1] Michard F, Chemla D, Teboul JL (2015). Applicability of pulse pressure variation: how many shades of grey?. Crit Care..

[2] Biais M, Ehrmann S, Mari A, Conte B, Mahjoub Y, Desebbe O (2014). Clinical relevance of pulse pressure variations for predicting fluid responsiveness in mechanically ventilated intensive care unit patients: the grey zone approach. Crit Care..

[3] Mahjoub Y, Pila C, Friggeri A, Zogheib E, Lobjoie E, Tinturier F (2009). Assessing fluid responsiveness in critically ill patients: false-positive pulse pressure variation is detected by Doppler echocardiographic evaluation of the right ventricle. Crit Care Med..

[4] Kim YK, Shin WJ, Song JG, Jun IG, Kim HY, Seong SH (2010). Effect of right ventricular dysfunction on dynamic preload indices to predict a decrease in cardiac output after inferior vena cava clamping during liver transplantation. Transplant Proc..

[5] Tavernier B, Makhotine O, Lebuffe G, Dupont J, Scherpereel P (1998). Systolic pressure variation as a guide to fluid therapy in patients with sepsis-induced hypotension. Anesthesiology..

